# Lethargy With Reversible Bilateral Basal Ganglia and Substantia Nigra Lesions in Anti-N-Methyl-D-Aspartate Receptor Encephalitis: A Case Report

**DOI:** 10.7759/cureus.113647

**Published:** 2026-07-30

**Authors:** Daisuke Abe, Masaoki Hidaka, Masaya Kumamoto, Yuka Kanazawa, Hitonori Takaba

**Affiliations:** 1 Department of Cerebrovascular Medicine and Neurology, Kyushu Central Hospital of the Mutual Aid Association of Public School Teachers, Fukuoka, JPN

**Keywords:** anti-n-methyl-d-aspartate receptor encephalitis, basal ganglia, cyclophosphamide pulse, ovarian teratoma, substantia nigra

## Abstract

Anti-N-methyl-D-aspartate receptor (anti-NMDAR) encephalitis is a neuroinflammatory disorder characterized by a broad spectrum of neuropsychiatric symptoms, and the optimal treatment strategy for atypical or refractory cases has not been well established. We report the case of a 26-year-old woman with anti-NMDAR encephalitis associated with an ovarian teratoma who presented with acute psychiatric and neurological manifestations. The patient underwent tumor resection followed by first-line immunotherapies, including corticosteroids, intravenous immunoglobulin, and plasma exchange. Because of persistent neurological symptoms, second-line immunotherapy with cyclophosphamide was initiated, leading to gradual clinical improvement. However, she subsequently developed lethargy and upper-limb tremors, accompanied by bilateral basal ganglia and substantia nigra abnormalities on MRI. Notably, both her clinical symptoms and radiological abnormalities improved following an additional course of cyclophosphamide treatment. This report highlights a rare clinical and radiological manifestation and suggests that additional immunotherapy may be effective for delayed neurological worsening.

## Introduction

Anti-N-methyl-D-aspartate receptor (anti-NMDAR) encephalitis is a rare neuroinflammatory disorder characterized by a broad spectrum of neuropsychiatric and neurological symptoms. Patients with anti-NMDAR encephalitis produce antibodies against the GluN1 subunit of the NMDAR in the cerebrospinal fluid (CSF) [1]. These antibodies induce internalization of NMDARs, resulting in disruption of NMDAR-mediated signaling. The disease typically follows a biphasic clinical course. In the first stage, patients experience a viral prodrome (fever, headache, or nausea), followed by psychiatric symptoms (hallucinations, mania, agitation, or disorganized thinking). Several weeks after symptom onset, neurological complications such as movement abnormalities, dysautonomia, hypoventilation, and seizures may develop. In the second stage, many acute symptoms gradually resolve, but patients may continue to experience behavioral alterations and cognitive and memory dysfunction during recovery [2,3].

Approximately half of patients with anti-NMDAR encephalitis have an associated tumor, most commonly an ovarian teratoma. These patients usually respond to a combination of tumor resection and immunotherapy. First-line immunotherapies include corticosteroids, intravenous immunoglobulin, and plasma exchange. Second-line therapies, such as rituximab or cyclophosphamide, are recommended for patients who do not respond to initial treatment [1,4]. MRI abnormalities involving the hippocampus and medial temporal lobes have been described during the first stage of the disease [5]. However, MRI lesions involving the basal ganglia and substantia nigra during the second stage of the disease have not been previously reported.

We report the case of a 26-year-old woman who developed acute psychiatric and neurological symptoms and was ultimately diagnosed with anti-NMDAR encephalitis associated with an ovarian teratoma. The patient underwent tumor resection on day 28, followed by a series of first-line therapies, including corticosteroids, intravenous immunoglobulin, and plasma exchange. As her neurological symptoms persisted, cyclophosphamide was introduced on day 59, leading to gradual clinical improvement. However, she subsequently developed lethargy and a bilateral upper-limb postural tremor of approximately 6-8 Hz, accompanied by distinct MRI abnormalities in the bilateral basal ganglia and substantia nigra identified on day 61. Both her clinical symptoms and radiological abnormalities improved following an additional course of cyclophosphamide therapy.

## Case presentation

A previously healthy 26-year-old woman presented with a headache and abnormal behavior, including purposeless wandering around a room. Before this episode, she had experienced a fever for one week. On neurological examination, she exhibited nuchal rigidity and disorientation to place, with a Glasgow Coma Scale (GCS) score of 14/15 (E4V4M6). Other neurological findings were normal. Lumbar puncture revealed mononuclear leukocytosis (43/µL; reference range: < 5/µL). Multiplex polymerase chain reaction testing of the CSF for meningitis was negative. Shortly after admission, the patient began shouting repetitively and was unable to follow simple instructions. The next day, she developed visual hallucinations and was observed talking to herself. On day five, she became unresponsive to external stimuli and developed generalized tonic-clonic seizures, with a GCS score of 3/15 (E1V1M1). Given the acute, progressive neuropsychiatric symptoms occurring in a young adult woman, anti-NMDAR encephalitis was suspected. Other differential diagnoses of acute psychosis include endocrine, neurologic, oncologic, and metabolic diseases [6].

CT of the pelvis revealed a left ovarian teratoma. Brain MRI revealed no apparent lesions. On day six, first-line therapy with intravenous methylprednisolone pulse therapy (IVMP, 1000 mg/day for three days) was initiated. However, the patient's clinical symptoms persisted. On day 14, she developed orofacial dyskinesia. On day 25, a cell-based assay confirmed the presence of anti-NMDAR antibodies in the CSF with a titer of 1:40 (reference range: negative or < 1:1). Other neuronal cell-surface antibodies against LGI1, CASPR2, AMPAR1/2, GABAA/B receptor, DPPX, and IgLON5, and paraneoplastic antibodies against Hu, Yo, Ri, Ma2, CV2/CRMP5, and amphiphysin were all negative. Accordingly, the patient was diagnosed with anti-NMDAR encephalitis associated with an ovarian teratoma. She underwent tumor resection, and the tumor was completely resected. On day 28, we initiated a second course of IVMP (1000 mg/day for three days), followed by intravenous immunoglobulins (0.4 g/kg/day for five days). On day 40, we performed plasma exchange (three sessions per week).

**Figure 1:**
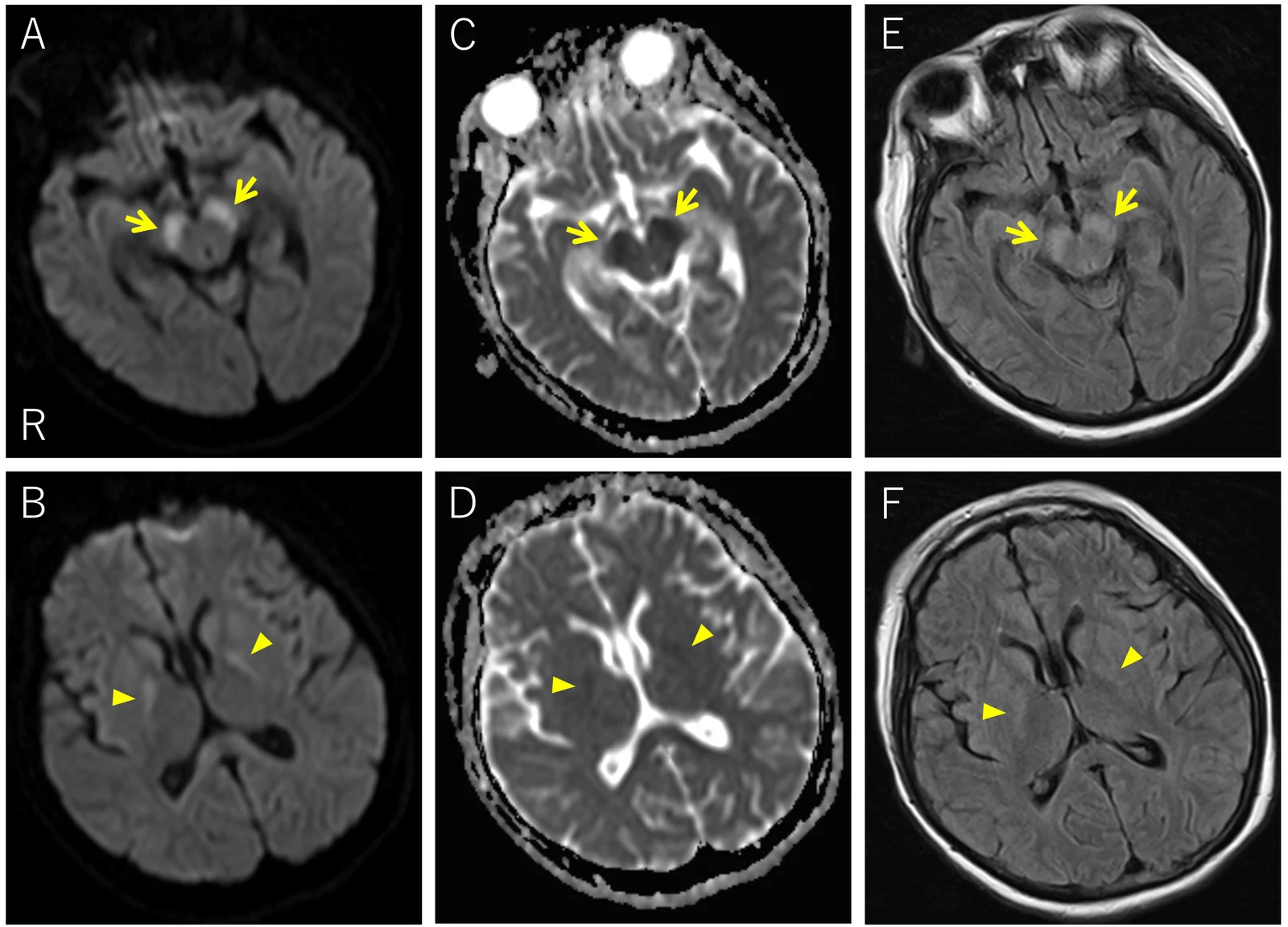
MRI findings on day 61 (A, B) Diffusion-weighted imaging (DWI) shows bilateral high signal intensities in the substantia nigra (A, yellow arrows) and the basal ganglia (B, yellow arrowheads). (C, D) The corresponding apparent diffusion coefficient (ADC) maps reveal decreased ADC values in the substantia nigra (C, yellow arrows) and the basal ganglia (D, yellow arrowheads), indicating restricted diffusion consistent with cytotoxic edema. (E, F) Fluid-attenuated inversion recovery imaging demonstrates mild hyperintensity in the same regions of the substantia nigra (E, yellow arrows) and the basal ganglia (F, yellow arrowheads) MRI: magnetic resonance imaging

Despite these treatments, the patient's neurological symptoms persisted. Therefore, cyclophosphamide (750 mg/m²) was initiated on day 59. Her tonic-clonic seizures and orofacial dyskinesia gradually resolved. On day 60, she began responding to stimuli and was able to nod when called by her name. However, she exhibited hypersomnolence and experienced only brief periods of wakefulness. Cranial nerve examination was unremarkable. Motor examination revealed mild weakness in all extremities (Medical Research Council grade 4/5) with disuse muscle atrophy. Sensory examination was normal. Deep tendon reflexes were preserved, and Babinski signs were absent bilaterally. She exhibited a bilateral upper limb postural tremor of approximately 6-8 Hz. No rigidity or resting tremor was observed. Brain MRI using diffusion-weighted imaging on day 61 revealed bilateral high-intensity signals in the basal ganglia and substantia nigra. These lesions showed reduced apparent diffusion coefficient values, suggesting reversible cellular edema (Figure 1).

**Figure 2:**
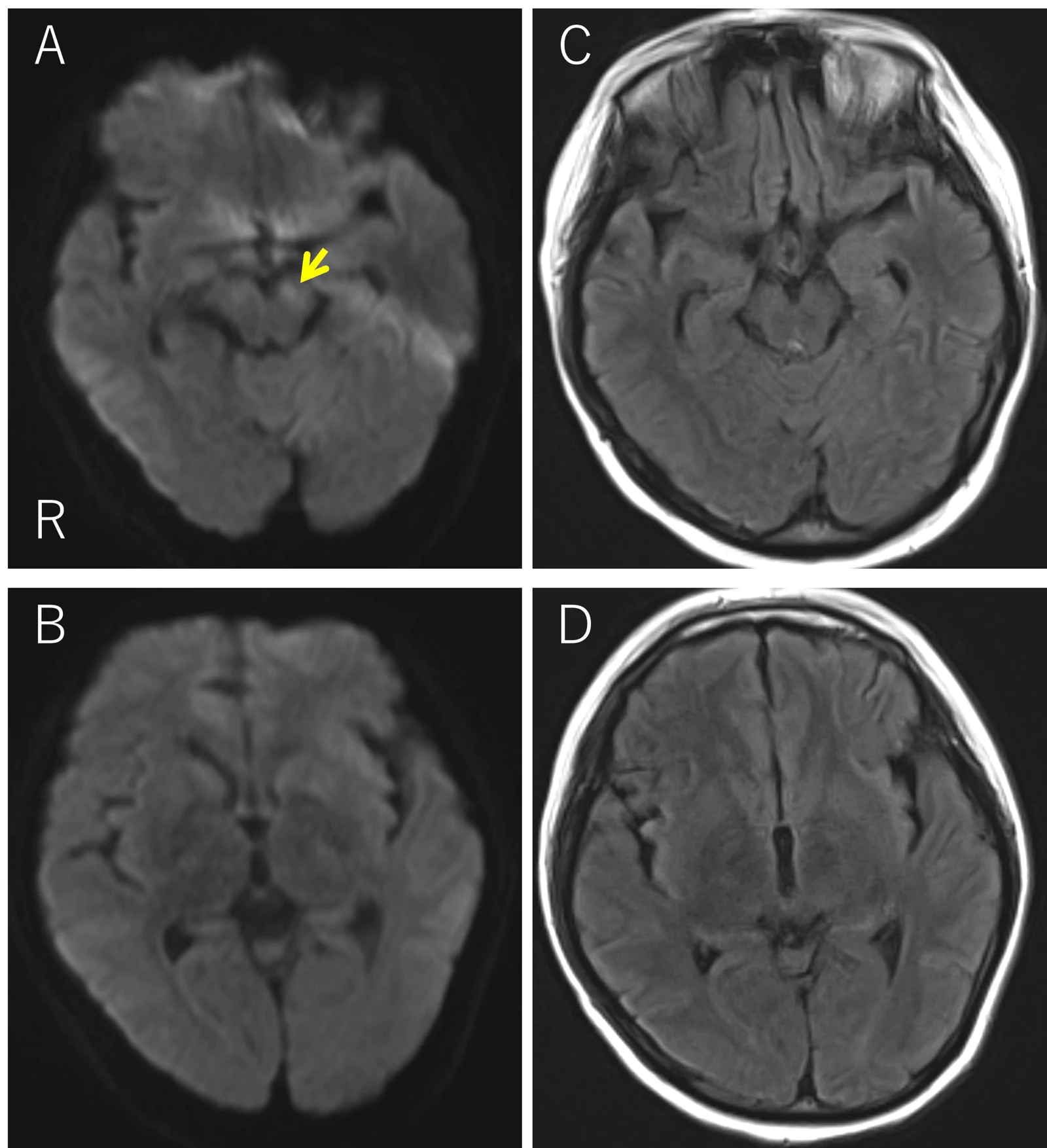
Follow-up MRI findings on day 92 (A, B) Diffusion-weighted imaging (DWI) demonstrated slight residual hyperintensity in the left substantia nigra (A, yellow arrowhead). The bilateral basal ganglia lesions observed on day 61 had completely resolved (B). (C, D) Fluid-attenuated inversion recovery images were normal MRI: magnetic resonance imaging

**Figure 3:**
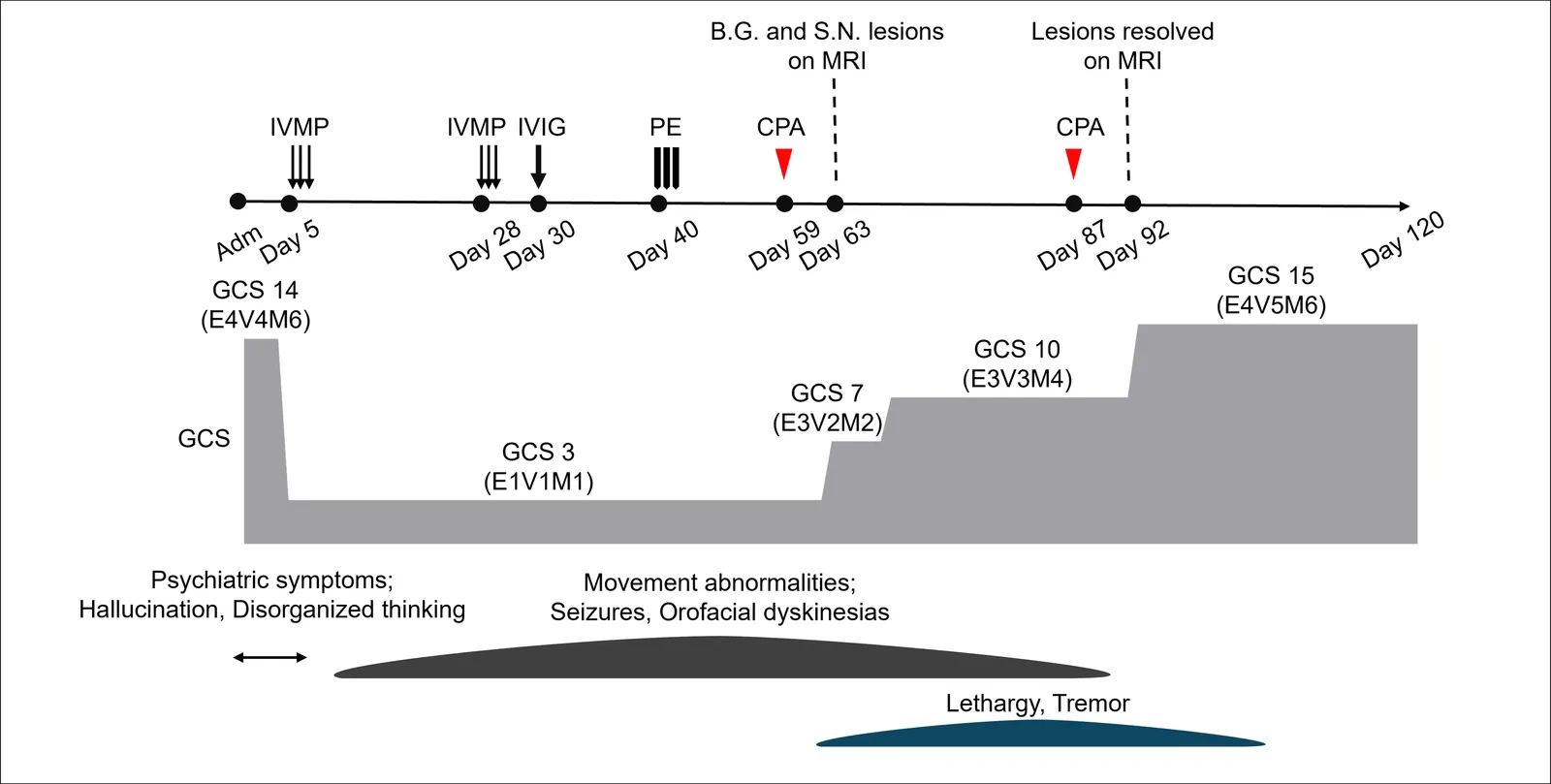
Clinical course of the patient Adm: admission; BG: basal ganglia; CPA: cyclophosphamide; GCS: Glasgow Coma Scale; IVIG: intravenous immunoglobulin; IVMP: intravenous methylprednisolone pulse; MRI: magnetic resonance imaging; PE: plasma exchange; SN: substantia nigra Figure created by the authors

Magnetic resonance angiography revealed no abnormalities. On day 80, anti-NMDAR antibodies were no longer detected in the CSF. Testing for aquaporin-4 antibodies and myelin oligodendrocyte glycoprotein antibodies was also negative (reference range: negative). Although the patient was able to follow simple instructions, hypersomnolence persisted. She opened her eyes in response to verbal stimuli and was able to briefly respond before returning to sleep. Therefore, a second course of cyclophosphamide (750 mg/m²) was administered on day 87. Her lethargy gradually resolved, and follow-up MRI on day 92 showed marked improvement of the lesions (Figure 2). The patient is currently undergoing rehabilitation and has experienced no clinical relapse through day 120 without maintenance therapy (Figure 3).

## Discussion

This report presents a rare case of a patient with anti-NMDAR encephalitis who developed lethargy and exhibited lesions in the bilateral basal ganglia and substantia nigra on MRI during the disease course. These clinical and radiological findings improved with immunotherapy. Approximately half of patients with anti-NMDAR encephalitis exhibit MRI abnormalities, most commonly in the hippocampus and medial temporal structures during the acute clinical phase [5]. To our knowledge, no previous case of anti-NMDAR encephalitis presenting with simultaneous lesions in the bilateral basal ganglia and substantia nigra during the subacute clinical phase has been reported. The radiologic differential diagnosis includes carbon monoxide poisoning, viral encephalitis (including Japanese encephalitis and West Nile encephalitis) [6], and neurodegenerative disorders, such as neurodegeneration with brain iron accumulation [7]. However, these conditions were considered unlikely based on the clinical presentation and disease course.

Although the exact pathophysiology underlying these MRI abnormalities remains unclear, two possibilities may be considered. First, immune-mediated mechanisms may have contributed to these lesions. Persistent immune-mediated inflammation despite CSF seronegativity may explain these lesions. Studies have demonstrated that anti-NMDAR antibody titers do not necessarily correlate with clinical symptoms [8]. Therefore, low-level or localized neuroinflammation may have persisted even after the CSF appeared seronegative. Moreover, alternative autoantibodies or novel immunologic targets may also be involved.

We investigated the possibility of an overlap syndrome by testing for other autoantibodies, such as aquaporin-4 and myelin oligodendrocyte glycoprotein antibodies [9,10], which were negative in our patient. Kreye et al. suggested that non-NR1 autoantibodies may contribute to central nervous system inflammation in patients with anti-NMDAR encephalitis [11]. Second, increased extracellular glutamate might induce neuronal injury. NMDAR antibodies are known to cause NMDAR internalization, which in turn increases extracellular glutamate concentration [12]. Increased extracellular glutamate may promote neuronal injury via oxidative stress. Neurons in the substantia nigra are exposed to high levels of oxidative stress because of their high basal metabolic demand [13].

Reactive oxygen species (ROS) are generated as a byproduct of ATP production, and substantia nigra dopaminergic neurons require exceptionally high levels of ATP compared with other neuronal populations. This is attributable to their large axonal arborization and autonomous pacemaking activity, both of which impose substantial energetic demands. In our case, we hypothesize that NMDAR internalization by anti-NMDAR antibodies might have increased extracellular glutamate, thereby imposing additional excitotoxic stress on neurons that were already under considerable metabolic demand. The combination of these intrinsic metabolic requirements and glutamate-mediated excitotoxicity may have amplified oxidative stress and contributed to the transient neuronal injury observed on MRI.

We speculate that the favorable clinical and radiological response to intensified immunotherapy suggests that immune-mediated mechanisms may have contributed to these lesions. In the management of anti-NMDAR encephalitis, clinicians often face difficulty determining whether residual symptoms warrant additional immunotherapy. In our patient, lethargy and tremor, accompanied by MRI lesions, prompted us to initiate additional immunotherapy. Our case suggests that additional immunotherapy may be considered in selected patients with persistent clinical and radiological findings suggestive of ongoing inflammation.

Titulaer et al. reported that, among those who failed to respond to first-line immunotherapy, receipt of second-line immunotherapy was associated with better functional outcomes than the absence of second-line immunotherapy [14]. As our patient did not respond to first-line immunotherapy, second-line treatment was required. Rituximab has become the preferred second-line therapy in many centers because of accumulating clinical experience and its favorable safety profile, although direct comparative evidence with cyclophosphamide remains lacking. Because rituximab was not available at our institution at that time, we administered cyclophosphamide as the second-line immunotherapy to avoid delaying treatment.

Notably, our patient developed marked lethargy in association with bilateral basal ganglia and substantia nigra lesions. The temporal association between the MRI abnormalities and the onset of lethargy raises the possibility that transient dysfunction of these structures contributed to impaired arousal. Although the precise mechanism remains unclear, both the clinical symptoms and MRI abnormalities improved following additional immunotherapy, suggesting that the lesions were functionally relevant.

This report has some limitations. First, patients who do not respond to first-line therapy four weeks after initiation are recommended to receive second-line therapy [14]. In our case, second-line therapy was initiated six weeks after the treatment onset. Therefore, it is possible that this delay in treatment may have contributed to the formation of the lesions. Second, metabolic or toxic etiologies cannot be completely excluded as potential causes of the atypical lesions.

## Conclusions

This case report highlights a rare presentation of anti-NMDAR encephalitis characterized by the development of bilateral basal ganglia and substantia nigra abnormalities during the disease course. The patient's neurological symptoms and imaging findings suggest that atypical MRI abnormalities may occur even after the acute phase of the disease.

## References

[1] Dalmau J, Armangué T, Planagumà J (2019). An update on anti-NMDA receptor encephalitis for neurologists and psychiatrists: mechanisms and models. Lancet Neurol.

[2] Endres D, Rauer S, Kern W (2019). Psychiatric presentation of anti-NMDA receptor encephalitis. Front Neurol.

[3] Zhao X, Teng Y, Ni J, Li T, Shi J, Wei M (2023). Systematic review: clinical characteristics of anti-N-methyl-D-aspartate receptor encephalitis. Front Hum Neurosci.

[4] Abboud H, Probasco J, Irani SR (2021). Autoimmune encephalitis: proposed recommendations for symptomatic and long-term management. J Neurol Neurosurg Psychiatry.

[5] Zhang T, Duan Y, Ye J (2018). Brain MRI characteristics of patients with anti-N-methyl-D-aspartate receptor encephalitis and their associations with 2-year clinical outcome. AJNR Am J Neuroradiol.

[6] Lersy F, Merdji H (2021). Substantia nigra involvement: an unusual finding in heat stroke. Neurology.

[7] Ishiyama A, Kimura Y, Iida A (2018). Transient swelling in the globus pallidus and substantia nigra in childhood suggests SENDA/BPAN. Neurology.

[8] Gresa-Arribas N, Titulaer MJ, Torrents A (2014). Antibody titres at diagnosis and during follow-up of anti-NMDA receptor encephalitis: a retrospective study. Lancet Neurol.

[9] Hacohen Y, Absoud M, Hemingway C (2014). NMDA receptor antibodies associated with distinct white matter syndromes. Neurol Neuroimmunol Neuroinflamm.

[10] Titulaer MJ, Höftberger R, Iizuka T (2014). Overlapping demyelinating syndromes and anti-N-methyl-D-aspartate receptor encephalitis. Ann Neurol.

[11] Kreye J, Wenke NK, Chayka M (2016). Human cerebrospinal fluid monoclonal N-methyl-D-aspartate receptor autoantibodies are sufficient for encephalitis pathogenesis. Brain.

[12] Manto M, Dalmau J, Didelot A, Rogemond V, Honnorat J (2010). In vivo effects of antibodies from patients with anti-NMDA receptor encephalitis: further evidence of synaptic glutamatergic dysfunction. Orphanet J Rare Dis.

[13] Trist BG, Hare DJ, Double KL (2019). Oxidative stress in the aging substantia nigra and the etiology of Parkinson's disease. Aging Cell.

[14] Titulaer MJ, McCracken L, Gabilondo I (2013). Treatment and prognostic factors for long-term outcome in patients with anti-NMDA receptor encephalitis: an observational cohort study. Lancet Neurol.

